# Obesity, Oxidative Stress, and Inflammation in Precocious Puberty: Do All Roads Lead to the Hypothalamus?

**DOI:** 10.3390/ijms27188159

**Published:** 2026-09-13

**Authors:** Teofana-Otilia Bizerea-Moga, Flavia Chișavu, Lazăr Chișavu, Laura Pitulice, Tudor Voicu Moga, Meda-Ada Bugi, Cornel Flavius Foghiș, Raluca Isac, Otilia Mărginean, Nicolae Constantin Balica

**Affiliations:** 1 Department XI of Pediatrics, First Pediatric University Clinic, Center for Research on Growth and Developmental Disorders in Children, ‘Victor Babeș’ University of Medicine and Pharmacy Timișoara, Eftimie Murgu Sq No. 2, 300041 Timișoara, Romania; bizerea.teofana@umft.ro (T.-O.B.-M.); cornel.foghis@umft.ro (C.F.F.); marginean.otilia@umft.ro (O.M.); 2 1st Pediatric Clinic, ‘Louis Țurcanu’ Children’s Clinical and Emergency Hospital, Iosif Nemoianu 2, 300011 Timișoara, Romania; bugi.ada@umft.ro; 3 Nephrology University Clinic, Centre for Molecular Research in Nephrology and Vascular Disease, ‘Victor Babeș’ University of Medicine and Pharmacy Timișoara, Eftimie Murgu Sq No. 2, 300041 Timișoara, Romania; farkas.flavia@umft.ro; 4 4th Pediatric Clinic, ‘Louis Țurcanu’ Children’s Clinical and Emergency Hospital, Iosif Nemoianu 2, 300011 Timișoara, Romania; isac.raluca@umft.ro; 5 Nephrology Clinic, ‘Pius Brînzeu’ County Emergency Clinical Hospital, Liviu Rebreanu 156, 300723 Timișoara, Romania; 6 Department of Chemistry, West University of Timişoara, Pestallozi 16, 300115 Timişoara, Romania; 7 Institute of Advanced Environmental Research, Oituz 4c, 300086 Timişoara, Romania; 8 Department VII of Internal Medicine, Gastroenterology University Clinic, Advanced Regional Research Center in Gastroenterology and Hepatology, ‘Victor Babeș’ University of Medicine and Pharmacy Timișoara, Eftimie Murgu Sq No. 2, 300041 Timișoara, Romania; moga.tudor@umft.ro; 9 Gastroenterology and Hepatology Clinic, ‘Pius Brînzeu’ County Emergency Clinical Hospital, Liviu Rebreanu 156, 300723 Timișoara, Romania; 10 Department of Pharmacy, University of Medicine and Pharmacy ‘Vasile Goldis’, 310025 Arad, Romania; 11 Ph.D. School Department, ‘Victor Babeș’ University of Medicine and Pharmacy Timișoara, Eftimie Murgu Sq No. 2, 300041 Timișoara, Romania; 12 Department XI of Pediatrics, Third Pediatric University Clinic, ‘Victor Babeș’ University of Medicine and Pharmacy Timișoara, Eftimie Murgu Sq No. 2, 300041 Timișoara, Romania; 13 Department IX, Otolaryngology University Clinic, OftalmoSensory-Tumor Research Center-ORL (EYE-ENT), ‘Victor Babeș’ University of Medicine and Pharmacy Timișoara, Eftimie Murgu Square 2, 300041 Timisoara, Romania; balica@umft.ro; 14 Otorhinolaryngology Clinic, Emergency City Hospital, 300054 Timisoara, Romania

**Keywords:** precocious puberty, hypothalamus, KNDy neurons, neuroinflammation, obesity, oxidative stress

## Abstract

Childhood obesity is consistently associated with earlier pubertal timing, particularly in girls, whereas its relationship with true central precocious puberty (CPP), defined by premature activation of the hypothalamic–pituitary–gonadal (HPG) axis, is less clearly established. An increased frequency of CPP diagnoses and referrals was reported during the COVID-19 pandemic, alongside changes in body weight, lifestyle, sleep, and psychosocial exposures. Human studies link excess adiposity to hyperleptinemia, insulin resistance, reduced adiponectin, altered sex-steroid bioavailability, and systemic low-grade inflammation, and associate these features with earlier pubertal development—consistently in girls, less so in boys. However, such associations do not establish that obesity directly induces premature hypothalamic activation. Mechanistic understanding of how these peripheral signals may influence pubertal timing derives predominantly from experimental models. The arcuate nucleus kisspeptin/neurokinin B/dynorphin (KNDy) network, a key component of the gonadotropin-releasing hormone (GnRH) pulse generator, interacts with hypothalamic metabolic circuits. In animal models of obesity and overnutrition, altered leptin and insulin signaling, mitochondrial reactive oxygen species generation, and activation of microglia and astrocytes remodel the mediobasal hypothalamus through inflammatory and stress-responsive pathways, including IKKβ/NF-κB and JNK signaling and altered Nrf2-mediated antioxidant defenses. At the molecular level, metabolic status interacts with the epigenetic machinery governing Kiss1 expression: in rodent models of overnutrition, accelerated loss of SIRT1-mediated repression at the Kiss1 promoter facilitates pubertal activation. Human genetic evidence establishes MKRN3 and DLK1 as causes of familial CPP, but evidence that obesity modifies these pathways to induce sporadic CPP is insufficient. Similarly, gut microbial metabolites have been linked experimentally to pubertal timing, and antioxidant micronutrients such as selenium, zinc, and vitamins C and E may be altered in pediatric obesity, yet a specific role in CPP remains unproven. Collectively, current evidence supports a working model in which metabolic, inflammatory, redox, glial, and epigenetic pathways may converge on hypothalamic reproductive circuits to influence pubertal timing in susceptible children. Direct evidence in children with CPP—of hypothalamic oxidative stress, glial activation, Nrf2 dysfunction, SIRT1 remodeling, or microbiome-mediated activation—remains limited or absent. Lifestyle optimization is appropriate for improving metabolic health in children with obesity, whereas antioxidant, micronutrient, and microbiome-targeted interventions should be considered investigational with respect to CPP. Longitudinal pediatric studies that distinguish earlier pubertal timing from true CPP, and that integrate metabolic phenotyping with validated measures of HPG-axis activation, are needed to test this framework.

## 1. Current State of Knowledge

Puberty is a critical period of human development that represents the shift from childhood to adolescence as a result of the integrated activation of the hypothalamic–pituitary–gonadal axis. The timing of reproduction is controlled by a complex interplay of genetic, epigenetic, metabolic, and environmental factors that together regulate the maturation of neuroendocrine pathways controlling reproduction [1,2,3,4]. The physiological mechanisms involved in pubertal onset are increasingly well understood, but the factors responsible for variation in pubertal timing remain an active area of research.

Puberty is initiated by the reactivation of pulsatile hypothalamic gonadotropin-releasing hormone (GnRH) secretion and subsequent activation of the hypothalamic–pituitary–gonadal (HPG) axis, leading to gonadal maturation, development of secondary sexual characteristics, the pubertal growth spurt, and acquisition of reproductive capacity. Clinically, pubertal onset in girls is defined by thelarche, corresponding to Tanner breast stage 2, and normally occurs between 8 and 13 years of age. In boys, pubertal onset is conventionally defined by testicular enlargement to a volume of ≥4 mL (or testicular length ≥ 2.5 cm), corresponding to Tanner genital stage 2, and normally occurs between 9 and 14 years of age. Pubic or axillary hair development reflects primarily adrenarche and, in isolation, does not establish activation of the HPG axis [4,5,6,7,8].

In recent decades, several epidemiological studies have reported a progressive reduction in the age of pubertal onset, particularly in girls, with contemporary cohorts undergoing breast development at younger ages than mid-20th-century reference populations [9]. This was particularly the case during the COVID-19 pandemic when pediatric endocrine centers worldwide noted a significant increase in referrals and diagnoses of central precocious puberty (CPP). A recent meta-analysis including 24,200 participants across 32 studies estimated that the odds of being diagnosed with precocious puberty (PP) among children referred for suspicion of the condition were nearly twice as high during the pandemic period as before (odds ratio = 1.96; 95% CI, 1.56–2.47; *p* < 0.001) [10]. Importantly, increased referrals or diagnoses of PP during this period do not necessarily indicate a corresponding increase in confirmed CPP or establish a causal effect of pandemic-related exposures on premature activation of the HPG axis. The reasons for the observed increase remain incompletely understood. Several lifestyle changes have been proposed as potential contributors, including increased use of electronic devices, sedentary behavior, higher body mass index (BMI) Z-scores, sleep disturbances, psychological stress, and reduced physical activity [10]. Concomitantly, alterations in the patterns of healthcare utilization and referral may have played a role in the observed increase in diagnoses.

The hypothalamus is essential for the coordination of the neuroendocrine events that initiate puberty by integrating signals related to energy balance, nutritional status, growth, and environmental conditions [1,4,7]. Childhood obesity has received particular attention because human studies consistently associate excess adiposity with earlier pubertal development, especially in girls, although the relationship is less consistent in boys [11,12,13,14]. Importantly, earlier breast development, pubarche, or advancement of other pubertal milestones in children with obesity does not necessarily demonstrate premature central activation of the HPG axis and should therefore be distinguished from true CPP [15,16,17]. Human studies of childhood obesity demonstrate alterations in metabolic and endocrine signals relevant to reproductive function, including hyperinsulinemia, hyperleptinemia, altered adipokine profiles, and chronic low-grade inflammation [11,18,19,20]. Experimental evidence provides potential mechanisms through which these obesity-associated signals may interact with hypothalamic reproductive circuits. In animal and cellular models, altered leptin and insulin signaling, inflammatory pathways, oxidative stress, and changes in adipokine signaling can modify hypothalamic networks involved in metabolic and reproductive regulation [2,18,21,22]. Among these networks, the kisspeptin/neurokinin B/dynorphin (KNDy) neuronal system is a key component of the gonadotropin-releasing hormone (GnRH) pulse generator, operating through coordinated signaling involving neurokinin B, dynorphin, kisspeptin, and their respective receptors [23]. These experimental observations provide biological plausibility for an interaction between metabolic status and the central regulation of pubertal timing. However, direct evidence that obesity-associated oxidative stress, neuroinflammation, or altered metabolic signaling within the hypothalamus causes premature activation of the GnRH pulse generator and CPP in children remains limited. The available findings therefore support a working hypothesis in which metabolic and inflammatory signals associated with excess adiposity may modify the hypothalamic environment and influence the timing or threshold of reproductive-axis activation in susceptible individuals, rather than an established causal pathway from obesity to CPP. The relative contributions of these mechanisms, their interactions with genetic and epigenetic susceptibility, and their relevance to girls and boys require further investigation. This narrative review summarizes current evidence on the hypothalamic mechanisms controlling pubertal timing, with particular emphasis on the KNDy neuronal network and its interactions with metabolic and environmental signals. It further evaluates the evidence linking obesity-associated metabolic, inflammatory, oxidative, and epigenetic alterations to earlier pubertal development and CPP, while distinguishing findings demonstrated in children from mechanisms established primarily in animal or cellular models and from pathways that remain hypothetical in humans.

## 2. Review Methodology

We searched PubMed/MEDLINE, Scopus, and Web of Science, supplemented by Google Scholar, from database inception through 2026 for peer-reviewed, English-language articles. Emphasis was placed on studies published from 2010 onward, which constitute the large majority of the cited literature, while seminal earlier work was retained where foundational (the earliest included reference dates to 1986; the search was performed on 1 June 2026). Search terms combined Medical Subject Headings and free-text keywords using Boolean operators, including “central precocious puberty,” “precocious puberty,” “pubertal timing,” “obesity,” “childhood/pediatric obesity,” “hypothalamus,” “arcuate nucleus,” “kisspeptin,” “KNDy neurons,” “GnRH,” “leptin,” “insulin,” “oxidative stress,” “reactive oxygen species,” “Nrf2,” “neuroinflammation,” “microglia,” “astrocytes,” “SIRT1,” “MKRN3,” “DLK1,” “selenium,” “selenoproteins,” “micronutrients,” “gut microbiota,” and “short-chain fatty acids,” in various combinations. Reference lists of retrieved articles and relevant reviews were hand-searched to identify additional sources.

Studies were selected on the basis of relevance to the mechanistic and clinical themes of the review, methodological soundness, and publication in peer-reviewed, indexed journals; non-indexed and predatory sources were excluded. The final synthesis draws on 150 references spanning 1986 to 2026, approximately 87% of which were published from 2010 onward. Where available, human studies were prioritized; mechanistic animal and cellular studies were included to illustrate candidate pathways and are identified as such throughout the text, in the evidence-level summary, and in the figures. Because this is a narrative synthesis, no protocol was registered and no formal risk-of-bias assessment or quantitative meta-analysis was performed; the resulting limitations, including the predominance of animal and cellular evidence over direct human data, are discussed in Section 8.

## 3. Hypothalamic Regulation of Pubertal Timing

### 3.1. GnRH Pulse Generator and KNDy Neurons

The onset of puberty is dependent upon the re-initiation of pulsatile gonadotropin-releasing hormone secretion after the relative quiescence of childhood. This transition is orchestrated by a highly interconnected hypothalamic network integrating developmental, hormonal, metabolic, and environmental signals. Within this network, neurons located in the arcuate nucleus (ARC) that co-express the neuropeptides kisspeptin, neurokinin B, and dynorphin, collectively known as KNDy neurons, are widely regarded as a key component of the GnRH pulse generator and play a central role in the regulation of reproductive function [1,24]. The principal hypothalamic neuronal populations integrating reproductive and metabolic signals are summarized in Table 1.

Experimental studies indicate that the three neuropeptides synthesized by KNDy neurons have complementary roles in the generation of pulsatile GnRH secretion. Kisspeptin is the main stimulatory input to GnRH neurons through binding to the kisspeptin receptor (KISS1R) and subsequent stimulation of GnRH release into the hypophyseal portal circulation [24,31]. Neurokinin B is proposed to mediate communication between neighboring KNDy neurons through tachykinin receptor 3 (NK3R) activity, which leads to synchronization of neuronal activity in the network; specifically, neurokinin B initiates and/or accelerates synchronized KNDy neuronal activity via stimulatory Gq-coupled NK3 receptors [24,25,26,30]. In contrast, dynorphin acts through κ-opioid receptors to terminate KNDy neuronal activity via inhibitory Gi-coupled κ-opioid receptors and thereby contribute to the maintenance of the rhythmic pattern of GnRH secretion. Experimental studies support a model in which the dynamic interplay between NKB-mediated excitation and dynorphin-mediated inhibition contributes to the oscillatory behavior of the GnRH pulse generator, although the precise organization and regulation of this network in humans remain incompletely defined [24,25,26]. The maturation of the KNDy network is a gradual developmental process, not an abrupt neuroendocrine switch. The restraint of GnRH secretion before puberty involves multiple regulatory mechanisms. Human genetic evidence identifies the imprinted gene Makorin ring-finger 3 (*MKRN3*) as an important inhibitor of pubertal initiation, with loss-of-function variants associated with CPP [35,36]. Experimental studies further demonstrate that epigenetic regulators, including Polycomb group proteins, repress *Kiss1* expression in hypothalamic ARC neurons and contribute to the prepubertal restraint of reproductive activation [37]. Across pubertal development, increasing stimulatory input to the GnRH neuronal network and attenuation of prepubertal inhibitory influences result in increased pulsatile GnRH secretion and maturation of reproductive function [1,24]. Experimental evidence further indicates that progressive epigenetic remodeling, including reversal of Polycomb-mediated repression of *Kiss1*, contributes to this transition [1,37]. Thus, pubertal onset is best understood as the progressive remodeling of interacting regulatory pathways rather than the activation of a single molecular trigger.

Experimental studies also implicate post-transcriptional regulation in the development and function of the KNDy network. Selective ablation of the Dicer enzyme in Kiss1-expressing neurons disrupts microRNA processing and causes delayed puberty and impaired fertility, with more pronounced effects in female mice. These changes are associated with altered KNDy neuronal projections and sensitivity to gonadal steroid feedback, supporting a role for microRNAs in the structural and functional maturation of hypothalamic reproductive circuits [23]. While these findings provide valuable mechanistic insight, their direct relevance to human pubertal disorders is still under investigation.

In addition to integration of endocrine and metabolic signals, the KNDy network appears to be responsive to external environmental cues. In an experimental rat model, early exposure to specific sensory stimuli activated cholinergic neurons in the medial amygdala and KNDy neurons in the ARC and was associated with earlier pubertal onset; inhibition of acetylcholine synthesis attenuated this effect [38]. These findings provide experimental evidence that sensory and environmental inputs can interact with reproductive circuits; however, whether an analogous mechanism contributes to CPP in children remains unknown.

### 3.2. Sexually Dimorphic Mechanisms

Sex differences are an important consideration when evaluating the relationship between adiposity and pubertal timing. Human observational studies demonstrate a more consistent association between higher BMI or adiposity and earlier pubertal development in girls, whereas findings in boys are more heterogeneous [12,13,39]. In girls, excess adiposity is associated particularly with earlier breast development and other markers of pubertal advancement [11,39]. However, these phenotypic changes do not necessarily demonstrate premature central activation of the hypothalamic–pituitary–gonadal axis [16,17]. In boys, the relationship between adiposity and pubertal timing appears less linear, and both earlier and delayed pubertal development have been reported across different degrees of excess weight and study populations [12]. These differences caution against extrapolating obesity-associated pubertal mechanisms identified predominantly in girls to boys.

Sexual dimorphism is also evident within the hypothalamic circuitry regulating reproductive function. Although arcuate KNDy neurons participate in the pulsatile regulation of GnRH secretion in both sexes [24], another population of kisspeptin neurons located in the anteroventral periventricular nucleus (AVPV) is markedly more prominent in females and mediates estrogen-positive feedback required for generation of the preovulatory luteinizing hormone (LH) surge [28,34,40]. Experimental studies demonstrate functional connectivity between AVPV kisspeptin neurons and GnRH neurons, with AVPV kisspeptin neurons synapsing onto GnRH neurons and exhibiting increased neuronal activation in response to the positive-feedback effects of estradiol [34,41,42]. Thus, the neural circuitry governing reproductive function shows important sex-specific organization in addition to the shared KNDy mechanisms controlling pulsatile GnRH secretion.

These neurobiological differences provide a plausible framework for sex-specific responses of the reproductive axis to metabolic and environmental signals. However, much of the detailed evidence concerning AVPV–kisspeptin–GnRH circuitry derives from experimental models, and it remains uncertain whether these sexually dimorphic mechanisms explain the stronger association between excess adiposity and earlier pubertal development, or the predominance of CPP observed in girls. Accordingly, sex should be considered an important biological modifier of the relationship between metabolic status and pubertal timing rather than assuming equivalent effects of obesity on hypothalamic reproductive activation in girls and boys.

### 3.3. Inhibitory and Stimulatory Pathways

In the prepubertal period, the hypothalamic–pituitary–gonadal axis is kept in a state of relative quiescence by the combined action of neurochemical inhibitory mechanisms and genetic factors [43]. During this period, pulsatile gonadotropin-releasing hormone secretion is markedly reduced. Experimental studies in primates have implicated inhibitory neurotransmitters, including γ-aminobutyric acid (GABA) [44] and neuropeptide Y (NPY) [45], in maintaining this prepubertal restraint. Experimental evidence further suggests that the KNDy network participates in the integration of GABAergic and NPYergic restraint before pubertal onset [44,46]. Human genetic studies provide direct evidence that disruption of inhibitory mechanisms controlling pubertal timing can cause CPP, particularly through abnormalities involving the imprinted genes *MKRN3* and *DLK1* [35,36,47,48]. *MKRN3* is an imprinted gene in the Prader–Willi syndrome region on chromosome 15q11.2 that encodes an E3 ubiquitin ligase. Its expression declines toward pubertal onset, and heterozygous loss-of-function variants represent the most frequent monogenic cause of familial CPP identified to date [35,36]. *DLK1* is a paternally expressed imprinted gene on chromosome 14q32 encoding a transmembrane protein involved in Notch signaling. Paternally inherited *DLK1* deletions and other abnormalities have been associated with non-syndromic familial CPP, with metabolic abnormalities reported in affected individuals [47,48]. These observations provide direct human evidence linking imprinted genetic regulation to premature HPG-axis activation; however, they do not establish that obesity or metabolic dysfunction modifies *MKRN3* or *DLK1* expression to cause sporadic CPP. Pubertal initiation reflects a gradual shift in the balance between inhibitory and stimulatory influences rather than the release of a single neuroendocrine brake. Experimental studies indicate that attenuation of tonic inhibitory signaling is accompanied by increasing excitatory inputs, including glutamatergic neurotransmission, insulin-like growth factor-1, and neurotrophic factors involved in neuronal connectivity and functional plasticity, including members of the EGF family produced by hypothalamic astrocytes [43,49,50]. Experimental models further implicate epigenetic remodeling in this developmental transition. During the juvenile period, Polycomb repressive complexes contribute to transcriptional repression of genes including *Kiss1* in the arcuate nucleus [37]. Remodeling of repressive chromatin marks, including H3K27 trimethylation and regulation by the H3K27 demethylase KDM6B, has been implicated in the *Kiss1–Tac3* transcriptional network associated with pubertal activation [51]. Reduction of these repressive epigenetic influences permits increased expression of *Kiss1* and related components of the KNDy network as puberty approaches [37,51]. Although these findings provide a mechanistic framework for developmental activation of the GnRH pulse generator, direct demonstration of Polycomb/KDM6B remodeling within the hypothalamus of children with CPP is lacking. Experimental evidence also supports an active role for glial cells in the neuroendocrine regulation of puberty. Astrocytes are now recognized as modulators of hypothalamic reproductive circuits, both by direct neuron-to-glia interactions and through the release of mediator molecules such as TGFα, neuregulins, prostaglandin E2, and neuroprogesterone, all of which can influence GnRH secretion [50]. Although these experimental findings establish glia as important components of reproductive neuroendocrine signaling, whether altered astrocyte function contributes directly to CPP in children remains unknown [50,52].

Pubertal onset therefore reflects the coordinated interaction of genetic, epigenetic, neurochemical, metabolic, and glial mechanisms rather than a single regulatory pathway. Together, these networks allow the hypothalamus to integrate developmental and physiological cues through changes in neuronal signaling, synaptic organization, and neuron–glia communication, enabling reproductive activation within an appropriate developmental context [1,52]. Human genetic causes such as *MKRN3* and *DLK1* abnormalities demonstrate that disruption of specific components of this regulatory architecture can result in CPP; however, the extent to which the other experimentally defined pathways contribute to idiopathic or obesity-associated CPP in children remains uncertain.

### 3.4. Metabolic Sensing by the Hypothalamus

The hypothalamus is an important regulator of energy balance and reproductive function [53,54]. Puberty and reproduction are metabolically expensive, and activation of the hypothalamic–pituitary–gonadal axis is tightly coupled to signals reflecting nutritional status and energy availability. This relationship is maintained in mammalian species and permits energetically expensive processes such as gametogenesis, pregnancy, and lactation to be conducted under favorable metabolic conditions. GnRH neurons express a limited set of metabolic hormone receptors, and therefore most of the information on the nutritional state of the body is integrated by other hypothalamic neurons that interact with the GnRH network [53]. The hypothalamus thus provides a major neuroendocrine interface between metabolism and reproduction [54].

A major site for this integration is the arcuate nucleus, which is located adjacent to the median eminence. Unlike most brain regions, the median eminence is a circumventricular structure characterized by fenestrated capillaries and relatively permissive access to circulating signals [55,56]. This specialized vascular structure enables communication between the peripheral circulation and hypothalamic neurons, allowing hormones, nutrients, and other metabolic signals in the circulation to reach neural circuits controlling energy homeostasis [56]. Permeable microvessels of the median eminence penetrate the ventromedial ARC, particularly in the vicinity of the stalk of the pituitary, providing an anatomical organization adapted to detect variations in the systemic metabolic environment [55].

The ARC therefore functions as an important chemosensory region integrating peripheral metabolic information with central neuroendocrine regulation. Leptin is one of the best-characterized metabolic hormones involved in pubertal regulation and appears to reach the hypothalamus in part via the median eminence. Access of circulating hormones to ARC neurons is not mediated solely by passive diffusion [57]. Tanycytes are specialized radial glia-like cells lining the floor of the third ventricle that actively regulate the blood–cerebrospinal fluid interface and participate in the transport of metabolic signals to hypothalamic neurons [56]. Experimental studies indicate that tanycytes can internalize circulating leptin through leptin receptor–epidermal growth factor receptor (LepR–EGFR)-dependent mechanisms and release it toward the cerebrospinal fluid through extracellular signal-regulated kinase (ERK)-dependent signaling [56,58,59]. However, impaired leptin transport is not consistently demonstrated in obesity. In diet-induced obese mice, Harrison et al. reported preserved leptin transport into the mediobasal hypothalamus, indicating that reduced hypothalamic leptin access is not required for the development of central leptin resistance [60]. Experimental studies further demonstrate that tanycytes dynamically regulate barrier permeability according to nutritional status. Fasting-induced vascular endothelial growth factor A (VEGFA)-dependent remodeling of the local vasculature alters access of circulating metabolites to ARC neurons, illustrating how hypothalamic metabolic sensing can depend on both vascular architecture and active cellular regulation [57,61]. The ARC contains closely linked metabolic and reproductive circuits. Experimental neuroanatomical and functional studies have characterized two major metabolically responsive neuronal populations located in close proximity to KNDy neurons [62]. One group consists of anorexigenic pro-opiomelanocortin (POMC) neurons that secrete α-melanocyte-stimulating hormone (α-MSH) and cocaine- and amphetamine-regulated transcript [63]. The other group is composed of orexigenic agouti-related peptide/neuropeptide Y (AgRP/NPY) neurons that stimulate food intake and decrease energy expenditure [33,64]. These neuronal populations have opposite responses to circulating metabolic signals such as glucose, leptin, and insulin [32,64]. The activity of POMC neurons and AgRP/NPY neurons is modulated by leptin and insulin under physiological conditions, and both cell populations are at the heart of the melanocortin system [64].

Evidence from animal models indicates that subsets of hypothalamic neurons involved in reproductive regulation can respond directly or indirectly to leptin, providing potential routes through which metabolic status can influence the reproductive axis [33,65]. In these studies, leptin deficiency is characterized by low Kiss1 expression in the ARC and failure of normal pubertal development, both of which are reversible by leptin replacement [65,66]. Taken together, these findings support the concept that leptin acts primarily as a permissive metabolic signal for reproductive maturation rather than as a primary trigger of puberty [65].

Preclinical neuroanatomical studies further demonstrate extensive bidirectional connectivity between reproductive and metabolic neuronal networks. KNDy neurons project to POMC and AgRP/NPY neurons. Arcuate Kiss1 neurons receive afferent input from a number of hypothalamic and extra-hypothalamic regions, including AVPV kisspeptin neurons, vasopressin neurons of the supraoptic and suprachiasmatic nuclei, thyrotropin-releasing hormone neurons of the paraventricular nucleus, as well as the amygdala, subfornical organ, ventral premammillary nucleus, and cortical areas. This extensive connectivity provides an anatomical substrate through which metabolic, hormonal, circadian, and environmental information may be integrated within reproductive neuroendocrine circuits [33,67]. Functional studies further support interactions between metabolic and reproductive neuronal populations. Kisspeptin stimulates POMC neurons and appears to inhibit AgRP/NPY neurons indirectly through GABAergic pathways [68,69], whereas AgRP neurons provide inhibitory input to Kiss1 neurons in the ARC and AVPV. Experimental activation of AgRP neurons inhibits Kiss1 neuronal activity, disrupts estrous cyclicity, and impairs fertility, consistent with suppression of reproductive function during negative energy balance. Conversely, α-MSH released from POMC neurons can increase arcuate Kiss1 neuronal activity and stimulate luteinizing hormone secretion in experimental models, supporting a role for melanocortin signaling in coupling energy availability to reproductive function [68]. During fasting or undernutrition, increased AgRP/NPY activity and reduced Kiss1 neuronal activity contribute to suppression of the reproductive axis [33,69]. Together, these experimental findings demonstrate reciprocal interactions between metabolic and reproductive hypothalamic circuits that allow reproductive function to adapt to changes in energy availability. The strategic anatomy of the median eminence and tanycytes, together with reciprocal interactions between KNDy and melanocortin circuits, provides a mechanistic framework through which metabolic signals may influence GnRH pulsatility and pubertal timing [54,56]. However, most of the detailed cellular and circuit-level mechanisms described in this section have been characterized in animal models. They establish biological plausibility for metabolic modulation of reproductive neuroendocrine function but do not demonstrate that obesity induces premature hypothalamic activation or CPP in children. Whether obesity-associated metabolic disturbances modify these pathways sufficiently to advance the GnRH pulse generator in susceptible children therefore remains to be established [54].

## 4. Obesity and Metabolic Signals in Earlier Pubertal Development

### 4.1. Adipose Tissue as an Endocrine Organ

Adipose tissue is now recognized as a metabolically active endocrine organ that secretes bioactive molecules known as adipokines [11,12,70]. Human observational studies have identified associations between excess adiposity and earlier pubertal development, particularly in girls [13,39]. Adipose tissue can influence reproductive physiology through endocrine, metabolic, and inflammatory signals relevant to the hypothalamic–pituitary–gonadal axis [11,18,71]. Increased adipose tissue in pediatric obesity is associated with significant changes in adipokine secretion, particularly higher levels of circulating leptin and lower concentrations of adiponectin [11,20,21]. Adiponectin has insulin-sensitizing and anti-inflammatory properties; reduced concentrations may therefore contribute to the insulin-resistant and pro-inflammatory metabolic phenotype associated with pediatric obesity [20,21,72]. Whether reduced adiponectin independently contributes to earlier activation of the reproductive axis in children remains uncertain. The leptin-to-adiponectin ratio has been proposed as a marker of adipose-tissue dysfunction and insulin resistance in pediatric obesity [20,73]. Its value as a biomarker for predicting earlier pubertal progression or CPP has not been established. Excess adiposity can also influence peripheral sex-steroid metabolism. Aromatase expressed in adipose tissue converts androgens to estrogens, and increased adipose mass has therefore been proposed to raise estrogen bioavailability [71]. In girls, this mechanism has been suggested to contribute to earlier breast development, but the evidence is not straightforward. Overweight and obese girls develop breast tissue at younger ages, yet do not consistently show higher circulating estrogen concentrations than normal-weight girls at the same breast stage, and a local rather than systemic source of estrogen has been proposed [74]. The relationship between adiposity, estrogen exposure, and early thelarche therefore remains incompletely resolved. Earlier thelarche, however, does not by itself demonstrate premature central activation of the HPG axis or establish CPP.

In obesity, adipose tissue, besides its endocrine role, also develops a chronic low-grade inflammatory phenotype [18]. Hypertrophic adipocytes and infiltrating immune cells secrete inflammatory mediators, including interleukin-6 and tumor necrosis factor-α [75]. Experimental studies indicate that obesity-associated inflammatory mediators can influence hypothalamic function through mechanisms involving blood–brain barrier regulation, glial activation, insulin signaling, and neuronal pathways controlling energy homeostasis [32,64,76]. Taken together, human observational evidence supports an association between excess adiposity, altered adipokine profiles, insulin resistance, increased peripheral sex-steroid bioavailability, and earlier pubertal development. Experimental studies provide biological plausibility for interactions between these metabolic and inflammatory signals and hypothalamic reproductive pathways. However, direct evidence that these obesity-associated changes lower the threshold of the GnRH pulse generator and cause CPP in children is lacking. They are therefore better considered components of a proposed multifactorial framework linking metabolic status to variation in pubertal timing rather than established causes of CPP.

### 4.2. Leptin and Insulin Signaling

Leptin is among the most well-characterized metabolic signals in pubertal maturation. Rather than acting as a primary trigger of puberty, leptin is considered a permissive metabolic signal indicating sufficient energy availability to support reproductive function [65,77]. Human genetic and interventional evidence strongly supports this permissive role. Congenital leptin deficiency and pathogenic leptin-receptor variants can be associated with hypogonadotropic hypogonadism and absent or delayed pubertal development, while leptin replacement in leptin-deficient patients can restore gonadotropin secretion and permit appropriately timed pubertal progression [78,79,80].

Children with obesity typically have elevated circulating leptin concentrations, reflecting increased adipose mass and, in many cases, leptin resistance [19,73]. In human observational studies, higher leptin concentrations have been associated with adiposity and earlier pubertal development, particularly in girls; however, these associations do not establish that hyperleptinemia causes CPP. Experimental studies suggest that leptin can modulate hypothalamic reproductive circuits, including kisspeptin-related pathways, directly or through intermediary neuronal populations [68,81,82]. In obesity, leptin’s metabolic and reproductive effects may not be affected to the same extent. Hypothalamic pathways controlling appetite commonly become resistant to leptin, whereas reproductive pathways may retain at least partial responsiveness to its permissive effects [77,82]. The mechanisms underlying this proposed selective leptin resistance remain incompletely understood. Human observational evidence has also linked insulin resistance and compensatory hyperinsulinemia with earlier pubertal development, particularly in the context of excess adiposity. Insulin acts at several levels of the reproductive axis, including the hypothalamus, pituitary, and gonads, where it maintains normal reproductive function under physiological conditions [83]. Experimental disruption of neuronal insulin receptor signaling results in hypothalamic dysregulation of luteinizing hormone and impaired gonadal function, supporting a physiological role for central insulin signaling in reproduction [59,84]. Hyperinsulinemia may influence reproductive physiology through peripheral effects on gonadal steroidogenesis and sex-steroid bioavailability, while experimental studies suggest additional effects on central reproductive pathways [84,85,86]. Animal studies have reported effects of increased insulin signaling on kisspeptin expression and markers of pubertal development [86]. The direct contribution of these mechanisms to CPP in children remains unknown.

Insulin resistance may also indirectly influence reproductive physiology through alterations in sex hormone-binding globulin (SHBG). Hyperinsulinemia is associated with reduced hepatic SHBG production and lower circulating SHBG concentrations, thereby increasing the proportion of bioavailable sex steroids [87]. Consequently, increased bioavailability of estrogens or androgens may contribute to earlier breast development, pubarche, or accelerated skeletal maturation [88]. These peripheral manifestations of pubertal advancement should not be interpreted as evidence of premature central GnRH activation in the absence of biochemical and clinical evidence of HPG-axis activation. Even though a transient decrease in insulin sensitivity is normal during puberty, children with obesity often enter puberty with pre-existing insulin resistance that can exacerbate the metabolic and endocrine changes associated with pubertal advancement.

Taken together, human studies support associations among excess adiposity, hyperleptinemia, insulin resistance, altered adipokine profiles, increased sex-steroid bioavailability, and earlier pubertal development. Experimental studies provide plausible mechanisms through which leptin and insulin signaling may interact with hypothalamic reproductive circuits. However, evidence that these metabolic abnormalities directly initiate premature GnRH secretion and cause CPP in children remains insufficient. The available data therefore support a multifactorial model of metabolic modulation of pubertal timing rather than attribution of CPP to any single adipocyte-derived or metabolic signal.

## 5. Neuroinflammation and Oxidative Stress: Converging Mechanisms in the Hypothalamus

### 5.1. Oxidative Stress and Hypothalamic Dysfunction

Oxidative stress has been recognized as a major component of metabolic dysfunction in obesity and increasingly as a contributor to hypothalamic dysfunction [22,89]. Oxidative stress arises when reactive oxygen species (ROS) production exceeds the capacity of endogenous antioxidant systems to maintain cellular redox homeostasis. At physiological concentrations, ROS participate in intracellular signaling, whereas excessive ROS production can damage lipids, proteins, nucleic acids, and mitochondrial components [90,91,92]. In obesity, chronic nutrient excess, mitochondrial stress, endoplasmic reticulum stress, and persistent low-grade inflammation can contribute to sustained oxidative imbalance [90,91,92].

Direct human evidence linking oxidative stress to CPP remains limited. A case–control study of girls with CPP, premature thelarche, and healthy controls evaluated circulating indices of oxidant–antioxidant balance, including total oxidant and antioxidant capacity, thiol–disulfide homeostasis, myeloperoxidase, catalase, and superoxide dismutase. The study identified alterations in systemic redox-related parameters among the groups; however, these circulating biomarkers do not constitute direct evidence of oxidative injury within the hypothalamus [93]. Thus, current pediatric evidence supports, at most, an association between CPP and peripheral redox markers rather than demonstration of hypothalamic oxidative stress as a causal mechanism.

In children with obesity, systemic oxidative stress and chronic low-grade inflammation are well-recognized components of metabolic dysfunction; however, circulating redox abnormalities should not be assumed to reflect oxidative injury within the hypothalamus [18,92]. Human studies therefore provide evidence for an obesity-associated pro-oxidative systemic environment, while direct demonstration that this environment alters hypothalamic reproductive circuits in children remains lacking.

**Figure 1:**
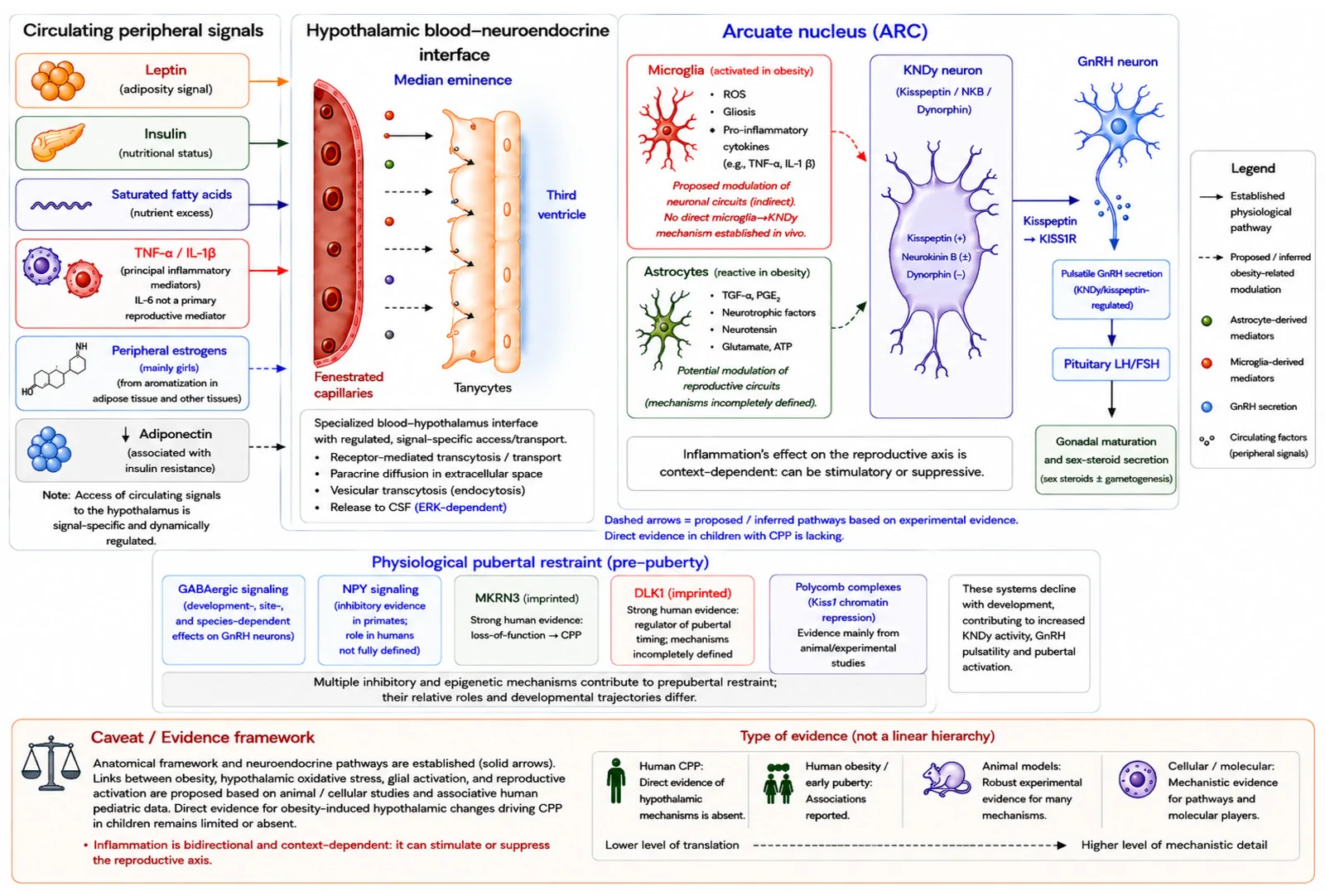
Proposed hypothalamic integration of obesity-related metabolic and inflammatory signals in pubertal regulation. Abbreviations: ARC, arcuate nucleus; ATP, adenosine triphosphate; CPP, central precocious puberty; CSF, cerebrospinal fluid; DLK1, delta-like non-canonical Notch ligand 1; ERK, extracellular signal-regulated kinase; FSH, follicle-stimulating hormone; GABA, γ-aminobutyric acid; GnRH, gonadotropin-releasing hormone; IL-1β, interleukin-1β; IL-6, interleukin-6; KISS1R, kisspeptin receptor; KNDy, kisspeptin/neurokinin B/dynorphin; LH, luteinizing hormone; MKRN3, makorin ring-finger protein 3; NKB, neurokinin B; NPY, neuropeptide Y; PGE_2_, prostaglandin E_2_; ROS, reactive oxygen species; TGF-α, transforming growth factor-α; TNF-α, tumor necrosis factor-α. This figure was created by the authors using OpenAI’s ChatGPT (GPT-5.6 Sol) and was reviewed and edited by the authors, who verified its scientific accuracy and take full responsibility for its content.

The hypothalamus may be particularly vulnerable to metabolic and oxidative stress because of the high energetic demands of hypothalamic neurons and its specialized exposure and responsiveness to circulating metabolic signals [56,57,89,94]. The ARC is located adjacent to the fenestrated capillaries of the median eminence and is therefore ideally situated to sense peripheral hormones and nutrients but is thus more exposed to changes in the systemic metabolic microenvironment [55,56,57,61]. The anatomical organization through which peripheral metabolic signals reach and influence the KNDy/GnRH network is summarized in Figure 1. In experimental models, nutrient excess promotes oxidative and inflammatory signaling within the hypothalamus, accompanied by neuronal stress and activation of microglia and astrocytes [18,22,89,95]. Experimental studies further implicate intracellular stress and inflammatory pathways, including IKKβ/NF-κB and JNK, in obesity-associated disruption of hypothalamic insulin and leptin signaling and metabolic regulation [96,97,98]. These findings establish mechanisms of obesity-associated hypothalamic dysfunction in experimental systems, but they do not demonstrate analogous changes within the hypothalamus of children with CPP.

Figure 1 illustrates how circulating peripheral signals reach the hypothalamus through the median-eminence blood–neuroendocrine interface and may modulate arcuate KNDy and GnRH neurons, alongside the physiological inhibitory and epigenetic mechanisms that restrain puberty before its onset. Established physiological pathways are shown as solid arrows and proposed obesity-related modulations as dashed arrows; open blue and red circles denote human and animal/cellular evidence, respectively.

Mitochondrial dysfunction represents another important component of cellular oxidative stress. Mitochondria are both major sources and targets of ROS, and sustained nutrient excess can increase mitochondrial oxidative burden and compromise cellular energy homeostasis [89,90,91,94,99]. In experimental models of hypothalamic metabolic dysfunction, these processes interact with inflammatory signaling and impaired insulin and leptin responses, thereby contributing to altered hypothalamic cellular function [22,95,96,98,100]. Direct evidence that mitochondrial oxidative dysfunction within reproductive hypothalamic neurons contributes to CPP in children is currently lacking. Evidence from animal studies indicates that ROS can directly modulate GnRH neuronal activity, although the direction of this effect is context-dependent. In mouse GnRH neurons, hydrogen peroxide altered neuronal excitability in a concentration- and developmental-stage-dependent manner, with higher concentrations predominantly suppressing excitability in adult neurons. These findings demonstrate that redox signals can act directly at the level of GnRH neurons but do not support a simple model in which oxidative stress uniformly stimulates reproductive activation. Rather, ROS appear to function as modulators of neuroendocrine signaling whose effects depend on concentration, developmental stage, and cellular context [101]. Whether analogous redox modulation contributes to premature GnRH activation in children with CPP remains unknown. Experimental studies have also examined environmental factors capable of modifying hypothalamic redox signaling. In rodent models, prolonged blue-light exposure has been associated with altered systemic oxidative-stress markers and earlier pubertal development [102]. However, there is currently no direct evidence that blue-light exposure causes CPP in children, and pandemic-era associations between screen exposure and increased CPP diagnoses remain observational and potentially confounded [103,104]. Oxidative stress and neuroinflammatory signaling are closely interconnected in experimental models. ROS can promote inflammatory cytokine production, whereas inflammatory signaling can further increase ROS generation through NADPH oxidase activation and mitochondrial dysfunction. This bidirectional interaction can establish a feed-forward cycle that amplifies cellular stress and disrupts hypothalamic metabolic signaling [90,91,96,98,99]. In obesity models, oxidative stress therefore interacts with inflammation, glial activation, and impaired metabolic signaling rather than operating as an isolated pathway [89,95,96,98]. These interactions provide biological plausibility for an indirect effect of metabolic oxidative stress on reproductive neuroendocrine circuits, but evidence that this process initiates CPP in children remains inferential.

Overall, the evidence supporting a role for oxidative stress in obesity-associated pubertal regulation is highly heterogeneous. Human studies in girls with CPP are limited to circulating redox markers and do not demonstrate hypothalamic oxidative injury [92,93]. Human obesity studies establish a systemic pro-oxidative and inflammatory milieu, whereas direct effects on hypothalamic reproductive pathways have been characterized predominantly in animal and cellular models [19,75,92,93,105]. Accordingly, oxidative stress should currently be regarded as a biologically plausible modifier of hypothalamic metabolic and reproductive signaling rather than an established causal mechanism of CPP. Future pediatric studies integrating validated endocrine phenotyping with longitudinal redox and metabolic biomarkers are required to determine whether oxidative imbalance precedes, accompanies, or results from premature HPG-axis activation.

### 5.2. Neuroinflammation and Microglial Activation

Direct evidence of hypothalamic microglial activation or neuroinflammation in children with CPP is currently lacking. In humans, evidence relevant to this pathway derives primarily from studies of obesity rather than CPP. Neuroimaging and neuropathological observations have identified findings compatible with hypothalamic injury or gliosis in individuals with obesity, paralleling changes observed in diet-induced obesity models [95,106]. These observations support the presence of obesity-associated hypothalamic alterations in humans but do not establish that such changes involve reproductive circuits or contribute to premature activation of the HPG axis. Experimental studies have provided considerably more detailed evidence, showing that chronic nutrient excess can induce inflammatory responses within the mediobasal hypothalamus [107,108]. Microglia, the resident immune cells of the central nervous system, normally contribute to tissue homeostasis, neuronal support, and synaptic remodeling, whereas metabolic excess can promote a reactive, pro-inflammatory microglial phenotype [107,108,109]. In experimental models of obesity, saturated fatty acids and nutrient excess activate inflammatory signaling within the mediobasal hypothalamus and promote the production of cytokines, prostaglandins, nitric oxide, and ROS [98,100,108,110,111]. Activated microglia can further increase ROS generation through mitochondrial dysfunction and NADPH oxidase activity, reinforcing the bidirectional interaction between oxidative stress and inflammation. Experimental studies also demonstrate remodeling of hypothalamic synaptic organization during diet-induced metabolic stress, including alterations in inputs to POMC and NPY neurons and reduced neuronal plasticity [112,113]. These findings establish a mechanistic link between nutrient excess, glial activation, and altered hypothalamic metabolic circuitry in experimental systems, but their relevance to reproductive circuits in children remains uncertain. Astrocytes also participate in the glial response to metabolic excess and can interact with microglia to amplify inflammatory signaling [114,115]. Experimental obesity models demonstrate accompanying changes in the hypothalamic cellular environment, including gliosis, macrophage infiltration, and synaptic remodeling. Importantly, some of these structural responses are sexually dimorphic: diet-induced obesity was associated with macrophage infiltration and reduced hypothalamic spine density in male but not female mice in one experimental study [95,113,116]. Thus, glial and structural responses to metabolic stress should not be assumed to occur identically in both sexes. The effects of inflammatory signaling on reproductive neuroendocrine pathways are complex and cannot be interpreted as uniformly stimulatory. Experimental studies show that inflammatory cytokines can act directly or indirectly on GnRH-related circuitry [117]. In rats, hypothalamic interleukin-1β and tumor necrosis factor-α mediate endotoxin-induced suppression of the reproductive axis [118]. At the cellular level, TNF-α impairs kisspeptin signaling in human GnRH primary neurons, while peripheral interleukin-1β reduces LH pulse frequency by inhibiting arcuate Kiss1 neuronal activation in female mice [119,120]. Thus, available mechanistic evidence demonstrates that acute or sufficiently intense inflammatory signaling can suppress, rather than activate, GnRH/LH function. Whether chronic, low-grade metabolic inflammation associated with obesity exerts qualitatively different effects on developing reproductive circuits remains unresolved. Independently of their uncertain role in pubertal initiation, hypothalamic inflammatory pathways are well characterized as contributors to altered energy homeostasis and central insulin and leptin resistance in experimental obesity models [22,96,98,114,121]. Engagement of IKKβ/NF-κB and JNK signaling in hypothalamic metabolic neurons can impair central nutrient and hormonal sensing [97,98,121], potentially reinforcing excess energy intake and metabolic dysfunction. Increased adiposity can in turn sustain systemic inflammation and oxidative stress, establishing reciprocal interactions among metabolic dysfunction, redox imbalance, and neuroinflammation [92,96,105,116,121]. In male mice, leptin and inflammatory factors have also been shown to interact with hypothalamic kisspeptin-related pathways, providing experimental evidence that metabolic and inflammatory signals can converge on reproductive circuitry [122]. However, these findings cannot be extrapolated directly to girls with CPP.

Collectively, these findings establish hypothalamic microglial activation and neuroinflammation as important components of experimental obesity-associated hypothalamic dysfunction. Human obesity studies provide indirect evidence of hypothalamic injury or gliosis, whereas direct evidence of microglial activation within reproductive hypothalamic circuits in children with CPP is absent. Moreover, experimental cytokine studies frequently demonstrate suppression of GnRH/Kiss1 signaling rather than reproductive activation. Chronic low-grade metabolic inflammation may therefore modify hypothalamic reproductive signaling in ways that differ from acute inflammatory responses, but this remains a working hypothesis rather than an established mechanism of obesity-associated CPP.

### 5.3. Astrocytes and Reproductive Neuroendocrine Signaling

Neurons are the main effector cells of the hypothalamic–pituitary–gonadal axis, but they are not alone. Astrocytes are increasingly recognized as active regulators of neuroendocrine function and pubertal development rather than passive structural support cells [50,123]. Astrocytes in the hypothalamus are involved in the maintenance of extracellular homeostasis, neurotransmitter recycling, and the supply of energy substrates to neurons for normal function. In addition, they link peripheral metabolic information to central neuroendocrine pathways. Because astrocytes closely surround blood vessels and the blood–brain barrier, they are ideally positioned to detect changes in circulating nutrients, hormones, and inflammatory mediators and rapidly relay this information to neighboring neurons [57,115,123].

Experimental studies support a direct role for astrocytes in the regulation of reproductive neuroendocrine signaling [50,52,123]. Astrocyte-derived neuroprogesterone can act on progesterone receptor-expressing kisspeptin neurons and facilitate GnRH/LH-surge circuitry [124,125]. Astrocytes also release gliotransmitters, including glutamate, ATP, D-serine, and prostaglandins, that modulate hypothalamic neuronal activity. In experimental preparations, astrocyte-derived prostaglandin E_2_ exerts a postsynaptic excitatory effect on GnRH neurons through EP2 receptor activation [50,126]. Experimental developmental studies further suggest that recruitment of astrocytes to GnRH neurons during infancy contributes to maturation of the GnRH neuronal network [127]. Obesity-associated astrocytic remodeling has been demonstrated primarily in experimental models and in studies of hypothalamic metabolic dysfunction. High-fat feeding induces reactive astrogliosis within the ARC–median eminence region, characterized by astrocytic hypertrophy and increased glial fibrillary acidic protein expression [95,106,115,128]. Experimental studies further show that saturated fatty acids and inflammatory signaling can promote astrocytic NF-κB activation and pro-inflammatory cytokine production [129]. Astrocytes may therefore participate, together with microglia, in sustaining an inflammatory and oxidative hypothalamic environment during metabolic excess [89,96,115]. However, direct evidence that obesity-induced astrocytic activation alters KNDy/GnRH function or contributes causally to CPP in children is currently lacking. Collectively, astrocytes are well-established components of hypothalamic reproductive signaling in experimental systems, and obesity can alter astrocytic structure and inflammatory function. These two observations provide biological plausibility for astrocyte-mediated modulation of pubertal neuroendocrine circuits; however, the intermediate steps linking obesity-associated astrogliosis to premature GnRH activation remain unproven in children with CPP.

### 5.4. Gut–Brain Axis and Microbial Metabolites

The microbiota–gut–brain axis has emerged as a potential interface between metabolic status and hypothalamic neuroendocrine regulation [130]. In children with obesity, alterations in gut microbial composition have been reported, together with increased fecal concentrations of short-chain fatty acids, including acetate, propionate, and butyrate [131]. However, the composition of the gut microbiota is highly variable across populations and methodologies, and specific taxonomic patterns should not be considered universal biomarkers of pediatric obesity. Importantly, these human studies demonstrate an association between obesity and altered gut microbial ecology, but they do not establish that microbiome changes influence hypothalamic reproductive pathways or cause CPP.

Experimental models provide mechanistic evidence that diet-induced changes in the intestinal microbiota can increase intestinal permeability and circulating lipopolysaccharide exposure, promoting peripheral immune signaling and central inflammatory pathways [130]. Through neural, endocrine, immune, and metabolic routes, microbial products and metabolites can influence hypothalamic function, including glial activation and neuroinflammatory signaling. These findings provide a plausible biological bridge between obesity-associated dysbiosis and central metabolic regulation, but direct evidence for analogous effects on reproductive hypothalamic circuits in children is lacking.

Short-chain fatty acids (SCFAs), particularly acetate, propionate, and butyrate, are among the best-characterized microbial metabolites relevant to gut–brain communication [130,132,133]. They contribute to intestinal epithelial energy metabolism, barrier integrity, and immune regulation and may also influence oxidative and inflammatory signaling [130]. In a female rat model of obesity-associated precocious puberty, supplementation with acetate, propionate, butyrate, or their combination was reported to delay pubertal progression and reduce hypothalamic GnRH-related signaling through the Kiss1–GPR54–PKC–ERK1/2 pathway [133]. Similarly, in an experimental model in which maternal high-fat feeding during lactation advanced female pubertal timing, microbial reconstitution was associated with partial normalization of pubertal development [134].

Together, these experimental findings indicate that the gut microbiota can modify pubertal timing in selected rodent models and provide mechanistic hypotheses involving microbial metabolites, inflammatory signaling, and hypothalamic reproductive pathways. However, no interventional evidence currently demonstrates that microbiome modification prevents, delays, or treats CPP in children. The gut–brain axis should therefore be regarded as an emerging and predominantly experimental component of the proposed obesity–puberty framework.

### 5.5. Antioxidant Defenses and Nutritional Modulation

The hypothalamus is constantly exposed to reactive oxygen species generated by normal cellular metabolism and therefore requires a proper antioxidant defense system to maintain neuronal activity and metabolic homeostasis [22,89,91,94]. One of the major protective mechanisms is the nuclear factor erythroid 2-related factor 2 signaling pathway, which regulates the expression of a large number of antioxidant and cytoprotective genes [22,135]. Upon activation, Nrf2 stimulates the synthesis of enzymes such as superoxide dismutase, catalase, glutathione peroxidase, heme oxygenase-1, and NAD(P)H quinone oxidoreductase 1. Collectively, these antioxidant systems limit excessive ROS accumulation and interact with inflammatory signaling pathways [22,135,136]. Chronic nutrient excess and persistent inflammatory and oxidative stress can impair these protective responses and contribute to hypothalamic metabolic dysfunction in experimental models [22,95,98]. Direct evidence of altered hypothalamic Nrf2 signaling in children with CPP is currently unavailable. Mechanistic evidence derives predominantly from animal models of metabolic dysfunction. In mice, conditional disruption of selenoprotein synthesis in hypothalamic cells increases oxidative stress, impairs leptin signaling, reduces POMC neuronal integrity, and promotes insulin and leptin resistance; enhancement of Nrf2 activity can ameliorate several of these abnormalities [22]. Experimental studies also demonstrate interactions between Nrf2 and glial inflammatory responses. In microglia, SIRT6 has been shown to stabilize Nrf2-dependent antioxidant transcription and attenuate high-fat-diet-associated inflammatory and metabolic dysfunction [137]. These findings establish Nrf2 as an important regulator of hypothalamic redox and inflammatory homeostasis in experimental obesity but do not demonstrate that Nrf2 dysfunction alters the GnRH pulse generator or causes CPP in children.

Nutritional status can influence systemic antioxidant capacity, and several micronutrients—including selenium, zinc, and antioxidant vitamins—contribute to redox homeostasis through enzymatic or direct antioxidant mechanisms [92,138,139]. In pediatric obesity, inadequate dietary quality and altered micronutrient status may coexist with chronic oxidative and inflammatory stress [115,138]. However, evidence that variation in these micronutrients directly modifies hypothalamic reproductive signaling in children is lacking.

Human pediatric evidence in this area primarily concerns obesity rather than CPP. In observational studies, children with excess weight have shown lower circulating selenium concentrations and glutathione peroxidase activity than normal-weight controls [140], and reduced circulating selenoprotein P has also been reported in children and adolescents with obesity [141]. Selenium status in children is also shaped by early-life and perinatal factors, including birth weight and gestational age [142]. Circulating selenium can be quantified by several analytical approaches, including spectrophotometric methods, hydride-generation atomic absorption or fluorescence spectrometry, and inductively coupled plasma mass spectrometry (ICP-MS) [143,144]. Other pediatric studies have associated lower vitamin C, vitamin E, or zinc status with adiposity, insulin resistance, or other metabolic abnormalities [115,138,139,145], while altered, variably reduced or compensatorily increased, activities of endogenous antioxidant enzymes have been described in children with obesity [138,139]. These findings indicate that altered micronutrient and antioxidant status can accompany pediatric obesity, but they do not demonstrate a causal relationship with pubertal timing or CPP.

Experimental evidence provides a more direct link between selenium biology and reproductive neuroendocrine function. In mice, central deficiency of SELENOT impaired gonadotropic-axis function, altered GnRH-related signaling, and affected reproductive phenotypes [146]. However, this model demonstrates a physiological role for a specific selenoprotein and should not be interpreted as evidence that selenium deficiency causes CPP in children.

Overall, human evidence supports an association between pediatric obesity and alterations in systemic antioxidant and micronutrient status, whereas direct evidence linking these abnormalities to CPP is absent. Experimental studies demonstrate that Nrf2-dependent antioxidant pathways and specific selenoproteins can influence hypothalamic metabolic and reproductive function, but these findings have not been translated into pediatric intervention studies. Evidence regarding antioxidant supplementation in childhood obesity currently concerns metabolic outcomes rather than the prevention or treatment of CPP [22,135,146,147]. Accordingly, a nutritionally adequate diet and evidence-based management of childhood obesity remain appropriate for overall metabolic health, but antioxidant, selenium, zinc, or other micronutrient supplementation cannot currently be recommended specifically to prevent or treat CPP in the absence of an established nutritional indication. Prospective pediatric studies are required to determine whether redox or micronutrient abnormalities merely accompany obesity or contribute independently to altered pubertal timing.

The translational strength of evidence supporting the principal metabolic, redox, inflammatory, glial, epigenetic, and gut–brain pathways discussed above is summarized in Table 2. Importantly, evidence linking a pathway to pediatric obesity or earlier pubertal timing should not be interpreted as evidence that the same pathway causes premature central HPG-axis activation.

## 6. Integrated Model: All Roads Lead to the Hypothalamus

Human evidence establishes the central importance of hypothalamic stimulatory and inhibitory pathways in pubertal regulation, but it does not support a single obesity-driven pathway leading to central precocious puberty (CPP). Among the strongest direct human CPP data are genetic abnormalities affecting physiological pubertal restraint. Loss-of-function variants in the imprinted gene *MKRN3* are an established cause of familial CPP [35,36], while paternally inherited abnormalities involving *DLK1* have also been associated with familial CPP and, in some affected individuals, metabolic abnormalities [47,48]. Conversely, disruption of stimulatory reproductive pathways produces the opposite phenotype: loss-of-function variants involving *KISS1R*, *KISS1*, *TAC3*, or *TACR3* can cause hypogonadotropic hypogonadism and failure of normal pubertal development [29,30,31]. Together, these human genetic findings demonstrate that the balance between stimulatory and inhibitory hypothalamic pathways is fundamental to pubertal timing. They do not, however, demonstrate that obesity modifies these pathways through oxidative stress, neuroinflammation, or epigenetic remodeling.

At the clinical level, human observational evidence consistently associates excess adiposity with earlier pubertal development, particularly in girls, whereas findings in boys are more heterogeneous [9,12,13,39]. Childhood obesity is accompanied by metabolic and endocrine changes, including hyperleptinemia, insulin resistance, altered adipokine profiles, and increased peripheral sex-steroid bioavailability, which may influence the developmental context in which puberty occurs [7,8]. However, earlier breast development or other advancement of pubertal milestones in children with obesity does not necessarily demonstrate premature central activation of the hypothalamic–pituitary–gonadal (HPG) axis. Accordingly, the association between obesity and earlier pubertal timing should not be regarded as equivalent to evidence that obesity directly causes CPP.

Mechanistic support for convergence of metabolic and reproductive signals within the hypothalamus derives predominantly from experimental models. Nutritional status can modify hypothalamic pathways involved in reproductive maturation, including leptin-sensitive neuronal circuits and KNDy-related signaling [24,33,53,54,68,69,77,81]. Experimental overnutrition and high-fat feeding can additionally induce hypothalamic oxidative stress, glial activation, impaired metabolic sensing, and inflammatory signaling [22,89,94,95,96,98,100,108,110,113,121,148]. These mechanisms provide biological plausibility for an interaction between metabolic excess and reproductive neuroendocrine circuitry but have not been demonstrated as a sequential pathway leading to CPP in children.

The relationship between inflammation and reproductive neuroendocrine function is particularly complex and should not be interpreted as uniformly stimulatory. In experimental models, hypothalamic interleukin-1β and tumor necrosis factor-α mediate endotoxin-induced suppression of the reproductive axis [117,118]. TNF-α has also been shown to impair kisspeptin signaling in human GnRH primary neurons [119], while peripheral interleukin-1β reduces LH pulse frequency through inhibition of arcuate Kiss1 neuronal activation in female mice [120]. Consistent with these inhibitory effects, chronic systemic inflammatory disease in children, exemplified by juvenile idiopathic arthritis, may be associated with delayed pubertal onset or slow pubertal progression [149]. Thus, the chronic low-grade inflammatory state accompanying obesity cannot currently be assumed to function as a direct stimulatory signal for GnRH activation. Whether chronic metabolic inflammation has distinct effects on the developing reproductive axis compared with acute or systemic inflammation remains unresolved.

Epigenetic regulation provides a more specific experimental mechanism through which nutritional status may influence pubertal timing. SIRT1, an NAD^+^-dependent deacetylase expressed in hypothalamic Kiss1 neurons, participates in the epigenetic repression of *Kiss1*. As physiological puberty approaches, SIRT1 occupancy at the *Kiss1* promoter decreases, facilitating the transition toward a transcriptionally permissive state. In experimental models, early overnutrition decreases SIRT1 content in Kiss1 neurons and accelerates removal of SIRT1-mediated repression at the *Kiss1* promoter, thereby increasing *Kiss1* expression and advancing pubertal development. Conversely, undernutrition increases hypothalamic SIRT1 levels, prolongs *Kiss1* repression, and delays puberty [37,53,149]. These findings establish a nutrient-sensitive SIRT1–*Kiss1* mechanism in experimental models; however, analogous SIRT1 remodeling has not been demonstrated directly in the hypothalamus of children with obesity-associated CPP. Likewise, the physiological decline in MKRN3-mediated pubertal restraint should not be interpreted as a demonstrated downstream effect of obesity.

At the cellular level, oxidative stress, neuroinflammation, glial activation, and impaired metabolic sensing are more appropriately considered interacting processes than successive steps in a causal cascade. Hypothalamic oxidative stress can contribute to leptin and insulin resistance, and experimental enhancement of Nrf2-dependent antioxidant signaling can ameliorate several of these metabolic abnormalities [22,135]. Glial antioxidant responses also interact with inflammatory signaling; for example, microglial SIRT6 can modulate Nrf2-dependent transcription in high-fat-diet-induced obesity [137]. These findings provide mechanistic evidence that redox and inflammatory pathways are interconnected within the metabolically stressed hypothalamus. Nevertheless, direct evidence demonstrating hypothalamic oxidative stress, microglial or astrocytic activation, Nrf2 dysfunction, or inflammation-driven KNDy activation in children with CPP remains limited or absent.

**Figure 2:**
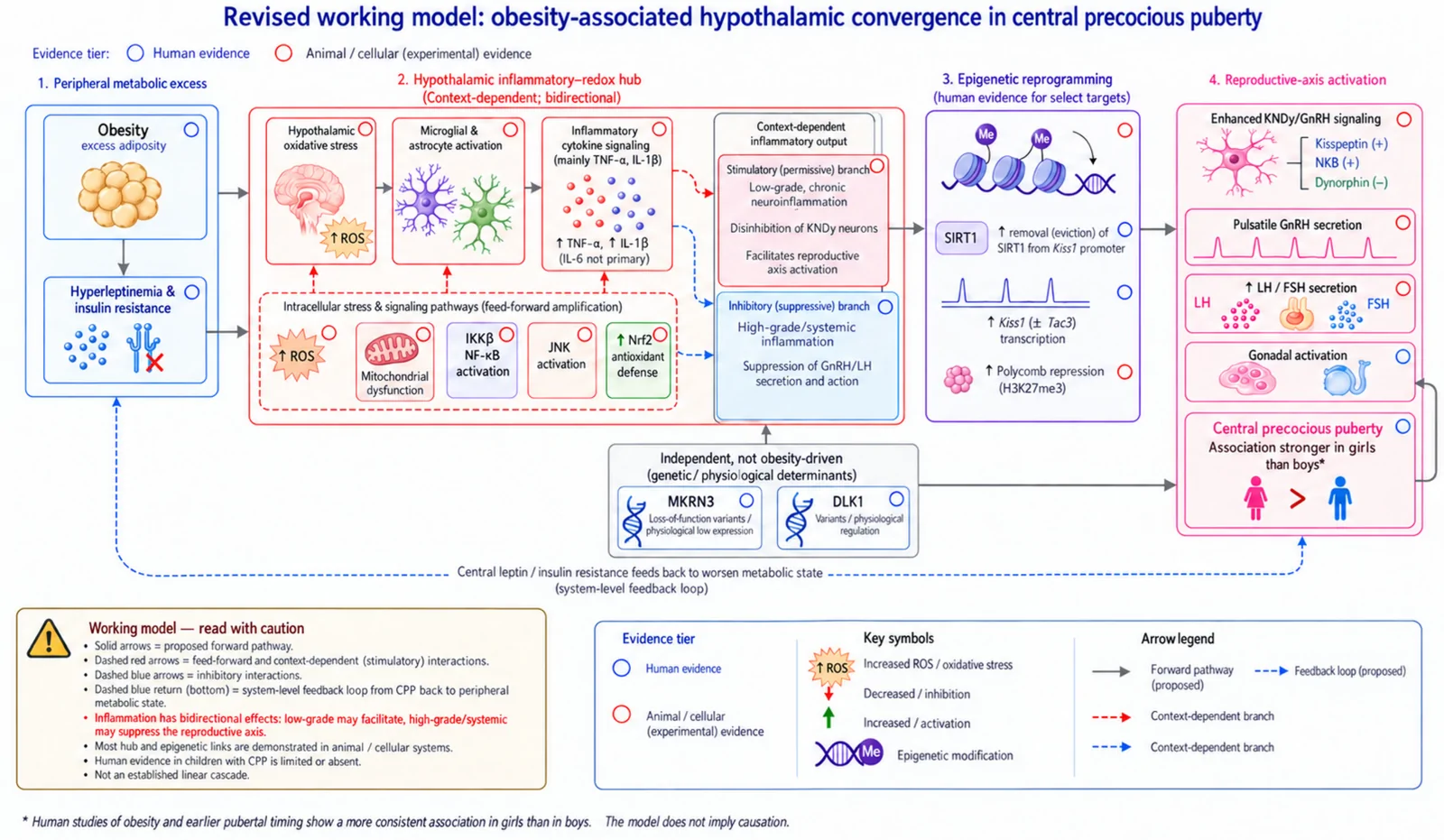
Proposed working model of obesity-associated hypothalamic convergence in central precocious puberty. Abbreviations: CPP, central precocious puberty; DLK1, delta-like non-canonical Notch ligand 1; FSH, follicle-stimulating hormone; GnRH, gonadotropin-releasing hormone; H3K27me3, histone H3 lysine-27 trimethylation; IKKβ, inhibitor of nuclear factor-κB kinase subunit β; IL-1β, interleukin-1β; IL-6, interleukin-6; JIA, juvenile idiopathic arthritis; JNK, c-Jun N-terminal kinase; Kiss1, kisspeptin gene; KNDy, kisspeptin/neurokinin B/dynorphin; LH, luteinizing hormone; MKRN3, makorin ring-finger protein 3; NF-κB, nuclear factor-κB; NKB, neurokinin B; Nrf2, nuclear factor erythroid 2-related factor 2; ROS, reactive oxygen species; SIRT1, sirtuin 1; Tac3, tachykinin 3 gene; TNF-α, tumor necrosis factor-α. This figure was created by the authors using OpenAI’s ChatGPT (GPT-5.6 Sol) and was reviewed and edited by the authors, who verified its scientific accuracy and take full responsibility for its content.

Taken together, the available evidence supports a proposed working model of hypothalamic convergence rather than an established linear obesity-to-CPP cascade (Figure 2). Genetic and developmental mechanisms establish the physiological balance between pubertal restraint and activation, while adiposity-related metabolic and hormonal signals may modify this regulatory environment. Experimental evidence suggests that oxidative stress, neuroinflammation, glial responses, impaired metabolic sensing, and SIRT1-dependent epigenetic regulation may interact through bidirectional and feed-forward relationships. In susceptible children, these processes could influence the timing or tempo of HPG-axis activation; however, whether they contribute causally to true CPP remains to be established. Their effects are also likely to depend on sex, developmental stage, genetic susceptibility, and the trajectory and duration of metabolic excess. The proposed model therefore distinguishes established human physiology and clinical associations from mechanisms demonstrated predominantly in animal or cellular systems and from links that remain inferential in humans.

Figure 2 presents a proposed working model in which peripheral metabolic excess (panel 1: obesity, hyperleptinemia, insulin resistance) may converge on hypothalamic reproductive circuits through a context-dependent inflammatory–redox hub (panel 2), epigenetic reprogramming (panel 3), and enhanced KNDy/GnRH signaling driving reproductive-axis activation (panel 4). Human evidence supports the peripheral associations (excess adiposity and earlier pubertal timing, particularly in girls) and the reproductive-axis physiology, whereas most links within the hypothalamic hub and the epigenetic step—hypothalamic oxidative stress, glial activation, neuroinflammation, Nrf2 signaling, and SIRT1-dependent *Kiss1* regulation—derive from animal or cellular studies. Inflammation is depicted as bidirectional: low-grade signaling may be permissive, whereas high-grade or systemic inflammation can suppress the axis. Genetic and physiological determinants of pubertal timing (MKRN3, DLK1) are shown as independent of obesity rather than as downstream consequences of it. Open blue and red circles denote human and animal/cellular evidence, respectively; solid arrows indicate proposed forward relationships, dashed red arrows indicate feed-forward and context-dependent interactions, and dashed blue arrows indicate inhibitory interactions and the system-level feedback loop. The diagram is a biologically plausible working framework and should not be interpreted as an established sequential pathway from obesity to CPP.

## 7. Clinical Implications and Future Directions

Recognition of the potential interaction between metabolic status and hypothalamic reproductive regulation has implications primarily for the comprehensive assessment of children with CPP rather than for the introduction of new mechanism-targeted therapies. GnRH agonists remain the established treatment for children with progressive CPP when treatment is clinically indicated. Their therapeutic objective is suppression of premature HPG-axis activation and prevention of the consequences of excessively early pubertal progression [8,15,17]. In children with concomitant overweight or obesity, weight management, adequate nutrition, physical activity, and treatment of associated metabolic abnormalities are appropriate components of general clinical care [15,19,70]. However, these interventions have not been demonstrated to reverse established CPP or to substitute for GnRH agonist treatment. Similarly, although micronutrient and antioxidant status may be altered in pediatric obesity, current evidence does not support selenium, zinc, antioxidant, or microbiome-directed supplementation specifically for the prevention or treatment of CPP [92,131,133,134,138,139,140,141,145,147].

At present, the diagnosis and assessment of CPP should continue to rely on established clinical and endocrine parameters, including age and tempo of pubertal progression, Tanner staging, growth velocity, bone-age advancement, basal and/or stimulated gonadotropin concentrations, and sex-steroid measurements, with imaging used according to the clinical context [4,5,8,16,17]. Metabolic evaluation may additionally be appropriate in children with overweight or obesity, but no obesity-related circulating or molecular biomarker has yet been validated for predicting CPP. The leptin/adiponectin ratio has been investigated in girls with established CPP in relation to subsequent weight gain [150], but its value for identifying children at risk of CPP has not been established. Proposed biomarkers related to hypothalamic inflammation and oxidative stress, as well as selenium status, gut microbial signatures, and epigenetic profiles, remain investigational rather than clinically validated biomarkers [37,53,72,92,93,131,133,134,140,141,150]. Longitudinal pediatric studies are therefore required to determine whether these measures provide predictive information beyond conventional auxological and endocrine assessment and, critically, whether they distinguish obesity-associated earlier pubertal timing from true premature central HPG-axis activation [8,14,15,17].

## 8. Limitations

This narrative review has several limitations. First, the available evidence is heterogeneous, and many of the proposed mechanisms linking obesity to hypothalamic pubertal activation—including oxidative stress, glial activation, Nrf2 signaling, SIRT1-mediated epigenetic regulation, and microbiome-related pathways—derive predominantly from animal or cellular models. Direct evidence of these processes in the hypothalamus of children with CPP is limited or absent. Second, human studies are largely observational and frequently address obesity-associated earlier pubertal timing rather than clinically confirmed CPP, limiting causal inference. Finally, differences in study populations, definitions of pubertal outcomes, and metabolic phenotyping restrict direct comparison across studies. Accordingly, the integrated model presented in this review should be interpreted as a biologically plausible working framework rather than an established causal pathway, and its principal mechanistic links require validation in prospective pediatric studies.

## 9. Conclusions

The evidence reviewed here supports the hypothalamus as a central integrator of the developmental, metabolic, hormonal, and environmental signals that regulate pubertal timing. In humans, genetic evidence establishes the importance of hypothalamic pathways governing pubertal restraint and activation, including *MKRN3*, *DLK1*, *KISS1/KISS1R*, and *TAC3/TACR3*. Clinical and epidemiological studies further demonstrate an association between excess adiposity and earlier pubertal development, particularly in girls. However, earlier pubertal timing in children with obesity should not be considered synonymous with central precocious puberty (CPP), and current human evidence does not establish obesity as a direct cause of premature hypothalamic–pituitary–gonadal axis activation.

Experimental studies provide a biologically plausible framework through which metabolic excess could influence hypothalamic reproductive regulation. Hyperleptinemia, insulin resistance, altered adipokine signaling, oxidative stress, mitochondrial dysfunction, and glial inflammatory responses can modify hypothalamic metabolic and reproductive circuits in animal and cellular models. Nutrient-sensitive epigenetic regulation, particularly SIRT1-dependent control of *Kiss1* expression, provides an additional mechanistic link between energy status and pubertal timing. Interactions involving Nrf2-dependent antioxidant defenses and the gut–brain axis further broaden this framework. Nevertheless, direct evidence of hypothalamic oxidative stress, microglial or astrocytic activation, Nrf2 dysfunction, SIRT1 remodeling, or microbiome-mediated activation of the reproductive axis in children with CPP remains limited or absent. These mechanisms should therefore be regarded as components of a proposed working model rather than an established causal cascade.

Accordingly, obesity-associated CPP is best considered within a multifactorial framework in which genetic susceptibility, developmental trajectories, sex-specific biology, metabolic status, and potentially environmental influences may interact to modify the timing and tempo of reproductive maturation. The available evidence is substantially stronger for an association between obesity and earlier pubertal development in girls than for obesity-induced CPP, while the relationship in boys remains less consistent. Oxidative stress and neuroinflammation may represent mechanistic interfaces between metabolic dysfunction and hypothalamic signaling, but whether they contribute causally to CPP in humans remains to be established.

These translational limitations also have important clinical implications. GnRH agonists remain the established treatment for progressive CPP when treatment is indicated. In children with overweight or obesity, optimization of weight status, dietary quality, physical activity, and associated metabolic abnormalities is appropriate for overall metabolic health, but these measures have not been demonstrated to reverse established CPP or replace GnRH agonist therapy. Likewise, antioxidant micronutrients, including selenium and zinc, and microbiome-targeted interventions should currently be regarded as investigational in relation to CPP rather than as established adjunctive treatments.

Future research should prioritize longitudinal pediatric studies capable of distinguishing obesity-associated earlier pubertal timing from true premature central activation of the HPG axis. Prospective studies integrating detailed pubertal phenotyping with metabolic, inflammatory, oxidative-stress, and potentially microbiome or epigenetic measures may clarify whether these pathways provide predictive information beyond established clinical and endocrine assessment. Ultimately, determining which experimentally identified mechanisms are operative in children—and whether any are modifiable—will be necessary before the proposed model of obesity-associated hypothalamic convergence can be translated into biomarker-guided prevention or individualized therapeutic strategies.

## Figures and Tables

**Table 1:** Principal hypothalamic neuronal populations involved in pubertal and metabolic regulation.

Neuronal Population	Principal Hypothalamic Location	Major Neurotransmitters/Neuropeptides	Principal Receptors or Signaling Targets Relevant to Reproductive Regulation	Major Physiological Function	Relevance to Metabolic–Reproductive Integration	Evidence Relevant to Human Puberty
KNDy neurons	Arcuate nucleus (ARC)	Kisspeptin; neurokinin B (NKB); dynorphin	Kisspeptin → KISS1R on GnRH neurons; NKB → NK3R/TACR3 within the KNDy network; dynorphin → κ-opioid receptors	Core component of the GnRH pulse generator. NKB promotes synchronization of KNDy activity, kisspeptin provides stimulatory input to GnRH neurons, and dynorphin contributes to pulse termination and rhythmicity.	Receive and integrate metabolic, hormonal and neuronal inputs. Interact bidirectionally with POMC and AgRP/NPY circuits and therefore provide a potential interface between energy status and reproductive activation.	Human genetic evidence involving KISS1/KISS1R and TAC3/TACR3 establishes the importance of this signaling system for normal pubertal activation; detailed circuit-level organization derives predominantly from experimental studies [24,25,26,27,28,29,30,31].
POMC neurons	Predominantly ARC	Pro-opiomelanocortin-derived peptides, particularly α-melanocyte-stimulating hormone (α-MSH); cocaine- and amphetamine-regulated transcript (CART)	Melanocortin signaling; responsive to leptin and insulin; α-MSH influences downstream melanocortin pathways and can modulate arcuate Kiss1 activity	Anorexigenic neurons promoting reduced food intake and increased energy expenditure; major component of hypothalamic energy sensing.	Activated by signals of energy sufficiency. Experimental studies show that α-MSH can increase arcuate Kiss1 neuronal activity and stimulate LH secretion, providing a mechanistic link between adequate energy availability and reproductive function.	Physiological relevance of melanocortin pathways to energy balance is established [32], but direct evidence that altered POMC signaling causes CPP in children is lacking.
AgRP/NPY neurons	ARC	Agouti-related peptide (AgRP); neuropeptide Y (NPY); GABA	Responsive to leptin and insulin; inhibitory inputs to Kiss1 neurons include AgRP-associated/GABAergic pathways	Orexigenic neurons that promote food intake and reduce energy expenditure; activity increases during negative energy balance.	Experimental activation inhibits arcuate and AVPV Kiss1 neuronal activity and can suppress reproductive function. Increased AgRP/NPY activity during fasting provides a mechanism coupling insufficient energy availability to suppression of the reproductive axis.	Circuit-level evidence is predominantly experimental; no direct evidence establishes AgRP/NPY dysregulation as a cause of CPP in children [33].
AVPV kisspeptin neurons	Anteroventral periventricular nucleus (AVPV), with marked sexual dimorphism	Kisspeptin	KISS1R on GnRH neurons; strongly regulated by estradiol-positive feedback	Particularly important in females for estrogen-positive feedback and generation of the preovulatory GnRH/LH surge rather than the basal pulsatile GnRH generator.	Provides sex-specific integration of reproductive hormonal signals and illustrates that hypothalamic responses relevant to puberty and reproduction are not identical in girls and boys.	Sexually dimorphic organization and GnRH connectivity are strongly supported experimentally [28,34]; their contribution to the stronger association between adiposity and earlier puberty/CPP in girls remains uncertain.

Abbreviations: AgRP, agouti-related peptide; ARC, arcuate nucleus; AVPV, anteroventral periventricular nucleus; CART, cocaine- and amphetamine-regulated transcript; GnRH, gonadotropin-releasing hormone; KISS1R, kisspeptin receptor; KNDy, kisspeptin/neurokinin B/dynorphin; LH, luteinizing hormone; NKB, neurokinin B; NK3R, neurokinin-3 receptor; NPY, neuropeptide Y; POMC, pro-opiomelanocortin.

**Table 2:** Translational evidence for proposed mechanisms linking obesity with hypothalamic regulation of pubertal timing and central precocious puberty.

Mechanism/Pathway	Human Observational Evidence	Human Interventional Evidence	Animal Evidence	Cellular/Molecular Evidence	Current Translational Interpretation
Leptin signaling	Children with obesity commonly have hyperleptinemia. Higher leptin concentrations are associated with adiposity and earlier pubertal development, particularly in girls. Congenital leptin deficiency demonstrates that adequate leptin signaling is necessary for normal reproductive maturation.	Yes, but in leptin deficiency—not obesity-associated CPP. Recombinant leptin treatment in congenital leptin deficiency can restore gonadotropin secretion and permit pubertal progression. No evidence demonstrates that reducing hyperleptinemia prevents or treats CPP in children with obesity.	Leptin deficiency reduces hypothalamic Kiss1 expression and impairs pubertal development; leptin replacement restores reproductive function. Leptin can modulate reproductive circuits directly and indirectly.	Leptin-sensitive neuronal circuits interact with KNDy, POMC and AgRP/NPY pathways. Selective resistance of metabolic versus reproductive pathways has been proposed experimentally.	Strong evidence that leptin is a permissive metabolic signal for reproduction; insufficient evidence that hyperleptinemia directly triggers CPP [65,66,68,70,72,73,77,78,80,81].
Insulin resistance/hyperinsulinemia	Insulin resistance and compensatory hyperinsulinemia accompany pediatric obesity and have been associated with earlier pubertal development. Hyperinsulinemia reduces hepatic SHBG production and may increase peripheral sex-steroid bioavailability.	No pediatric intervention has established that improving insulin resistance prevents or reverses CPP.	Experimental disruption of neuronal insulin signaling impairs gonadotropin secretion and reproductive maturation; increased insulin signaling can modify kisspeptin-related pathways and pubertal markers.	Insulin signaling interacts with hypothalamic metabolic neurons and reproductive pathways; peripheral actions also affect gonadal steroidogenesis and SHBG.	Established metabolic association and physiological role; direct causal contribution to premature central GnRH activation in children remains unproven [82,83,84,85,86,87,88].
Oxidative stress/mitochondrial ROS	Pediatric CPP data are limited to circulating redox markers. A case–control study in girls demonstrated differences in systemic oxidant/antioxidant indices, but not hypothalamic oxidative injury. Pediatric obesity is associated with systemic oxidative stress.	No intervention has demonstrated that antioxidant treatment prevents or treats CPP.	Diet-induced obesity and nutrient excess produce hypothalamic oxidative stress and mitochondrial dysfunction. Experimental ROS exposure can modify GnRH neuronal excitability, with effects dependent on concentration and developmental context.	ROS interact bidirectionally with mitochondrial dysfunction, NADPH oxidase activity, inflammatory signaling, insulin resistance and leptin resistance.	Biologically plausible modifier; direct hypothalamic oxidative stress has not been demonstrated in children with CPP and should not be regarded as an established causal mechanism [22,89,92,93,94,99,101].
Microglial activation/neuroinflammation	Human obesity studies provide indirect evidence compatible with hypothalamic injury or gliosis. Direct demonstration of hypothalamic microglial activation in children with CPP is absent.	No human intervention targeting hypothalamic inflammation has been shown to modify CPP.	High-fat feeding and nutrient excess induce mediobasal hypothalamic microglial activation, cytokine production, ROS generation and synaptic remodeling. Some responses are sexually dimorphic.	IKKβ/NF-κB, JNK, cytokine and NADPH-oxidase pathways link inflammatory signaling to altered hypothalamic metabolic sensing. Importantly, IL-1β and TNF-α can suppress rather than stimulate Kiss1/GnRH/LH signaling in experimental systems.	Strong evidence in experimental obesity, limited indirect human obesity evidence, but no direct evidence that microglial activation drives CPP. Inflammatory effects on reproductive signaling are context-dependent and not uniformly stimulatory [18,95,96,98,100,106,107,108,110,113,116,117,118,119,120,122,148].
Astrocytic activation/astrogliosis	No direct demonstration that hypothalamic astrocytic activation contributes to CPP in children. Human evidence is mainly indirect and derived from obesity-related hypothalamic alterations.	None relevant to CPP.	High-fat feeding causes reactive astrogliosis in the ARC–median eminence region. Astrocytes participate in obesity-associated inflammatory and oxidative responses.	Astrocytes modulate reproductive neurons via TGFα, neuregulins, neuroprogesterone, PGE_2_, glutamate, ATP and other gliotransmitters. PGE_2_ can excite GnRH neurons experimentally.	Astrocytes are established experimental regulators of reproductive neuroendocrine signaling, but the proposed obesity → astrogliosis → KNDy/GnRH → CPP sequence remains unproven in humans [21,50,95,106,115,123,124,125,127,128,129].
Nrf2-dependent antioxidant defenses	No direct evidence of altered hypothalamic Nrf2 signaling in children with CPP.	No Nrf2-targeted pediatric CPP intervention studies.	Manipulation of Nrf2-dependent antioxidant signaling modifies hypothalamic oxidative stress, leptin resistance and insulin resistance in obesity models.	Nrf2 regulates antioxidant/cytoprotective enzymes and interacts with inflammatory pathways; microglial SIRT6 can modulate Nrf2-dependent transcription.	Mechanistically strong experimental evidence for hypothalamic redox homeostasis; no evidence that Nrf2 dysfunction causes CPP in children [22,89,93,94,135,136,137].
SIRT1–Kiss1 epigenetic regulation	Direct hypothalamic SIRT1 remodeling has not been demonstrated in children with obesity-associated CPP.	None.	Early overnutrition decreases SIRT1 content in Kiss1 neurons and accelerates removal of SIRT1-mediated repression from the Kiss1 promoter, increasing Kiss1 expression and advancing pubertal development. Undernutrition produces the opposite pattern.	SIRT1 acts as a nutrient-sensitive epigenetic regulator of Kiss1 transcription and links energy status with pubertal timing.	Compelling nutrient-sensitive mechanism in experimental models, but its role in human obesity-associated CPP remains inferential [37,53].
MKRN3 and DLK1-mediated pubertal restraint	Strong direct human evidence for CPP. Loss-of-function MKRN3 variants and paternally inherited DLK1 abnormalities are established genetic causes of familial CPP; metabolic abnormalities have been described in some individuals with DLK1 abnormalities.	Not applicable as a metabolic intervention pathway.	Experimental studies support developmental regulation of hypothalamic inhibitory mechanisms, but the key relevance here derives from human genetics.	MKRN3 and DLK1 participate in pathways restraining or regulating pubertal activation; their normal developmental regulation forms part of the pubertal “brake.”	Established human CPP biology, but there is no evidence that obesity induces sporadic CPP by causing MKRN3 decline or modifying DLK1. These pathways must not be presented as demonstrated downstream consequences of obesity [35,36,47,48].
Gut microbiome/SCFAs	Children with obesity show differences in microbial composition/diversity and SCFA profiles, but findings vary across populations. Human data do not demonstrate microbiome-mediated hypothalamic activation or CPP.	None for CPP. No pediatric trial demonstrates that probiotics, prebiotics, SCFAs or other microbiome modification prevents, delays or treats CPP.	In female rodent models, SCFA supplementation can delay obesity-associated pubertal progression and modify hypothalamic Kiss1–GPR54–PKC–ERK1/2 signaling; microbial reconstitution has partially normalized advanced pubertal timing in another model.	Microbial metabolites and products can affect intestinal permeability, immune signaling, systemic inflammation, glial activation and hypothalamic signaling.	Emerging and predominantly experimental. A microbiome-driven mechanism for human CPP has not been established [130,131,132,133,134].
Micronutrients/selenium and antioxidant nutrients	Altered selenium, selenoprotein P, zinc, vitamins C/E and antioxidant-enzyme activity have been reported in pediatric obesity. These findings concern obesity/metabolic phenotype rather than established CPP causation.	Antioxidant supplementation studies in pediatric obesity address metabolic outcomes. **No intervention demonstrates prevention or treatment of CPP.**	Experimental selenoprotein deficiency can alter hypothalamic metabolic function; central SELENOT deficiency affects GnRH-related signaling and gonadotropic function in mice.	Selenium-containing proteins and antioxidant micronutrients contribute to redox homeostasis and interact with oxidative and inflammatory pathways.	**Nutritional adequacy is clinically appropriate, but micronutrient supplementation cannot currently be recommended specifically for CPP except for an independent nutritional indication** [92,138,139,140,141,145,147].

Evidence categories refer to the type of evidence available for the indicated pathway and should not be interpreted as equivalent levels of support for obesity-induced CPP. Human evidence may relate to established CPP, earlier pubertal timing, or pediatric obesity, as specified in each cell. Direct evidence of hypothalamic oxidative stress, microglial or astrocytic activation, Nrf2 dysfunction, SIRT1 remodeling, or microbiome-mediated reproductive activation in children with CPP remains limited or absent. Abbreviations: ARC, arcuate nucleus; CPP, central precocious puberty; IKKβ, inhibitor of nuclear factor κB kinase β; JNK, c-Jun N-terminal kinase; Nrf2, nuclear factor erythroid 2-related factor 2; ROS, reactive oxygen species; SCFA, short-chain fatty acid; SHBG, sex hormone-binding globulin.

## Data Availability

The original contributions presented in this study are included in the article. Further inquiries can be directed to the corresponding authors.

## Abbreviations

The following abbreviations are used in this manuscript:

AgRP	agouti-related peptide
ARC	arcuate nucleus
ATP	adenosine triphosphate
AVPV	anteroventral periventricular nucleus
BMI	body mass index
CART	cocaine- and amphetamine-regulated transcript
CPP	central precocious puberty
DLK1	Delta-like non-canonical Notch ligand 1
EGF	epidermal growth factor
EGFR	epidermal growth factor receptor
EP2	prostaglandin E2 receptor subtype 2
ERK	extracellular signal-regulated kinase
ERK1/2	extracellular signal-regulated kinases 1 and 2
FSH	follicle-stimulating hormone
GABA	γ-aminobutyric acid
GnRH	gonadotropin-releasing hormone
GPR54	G protein-coupled receptor 54
HPG	hypothalamic–pituitary–gonadal
IKKβ	inhibitor of nuclear factor κB kinase β
IL-1β	interleukin-1β
IL-6	interleukin-6
JNK	c-Jun N-terminal kinase
KDM6B	lysine demethylase 6B
KISS1R	kisspeptin receptor
KNDy	kisspeptin/neurokinin B/dynorphin neurons
LepR	leptin receptor
LH	luteinizing hormone
MKRN3	Makorin ring-finger protein 3
NADPH	reduced nicotinamide adenine dinucleotide phosphate
NF-κB	nuclear factor κB
NKB	neurokinin B
NK3R	neurokinin-3 receptor
NPY	neuropeptide Y
Nrf2	nuclear factor erythroid 2-related factor 2
PKC	protein kinase C
POMC	pro-opiomelanocortin
PP	precocious puberty
ROS	reactive oxygen species
SCFA	short-chain fatty acid
SELENOT	selenoprotein T
SHBG	sex hormone-binding globulin
SIRT1	sirtuin 1
SIRT6	sirtuin 6
TAC3	tachykinin precursor 3
TACR3	tachykinin receptor 3
TGFα	transforming growth factor α
TNF-α	tumor necrosis factor α
VEGFA	vascular endothelial growth factor A
α-MSH	α-melanocyte-stimulating hormone
